# Cortical interneurons in schizophrenia – cause or effect?

**DOI:** 10.3325/cmj.2023.64.110

**Published:** 2023-04

**Authors:** Matija Vid Prkačin, Ivan Banovac, Zdravko Petanjek, Ana Hladnik

**Affiliations:** Department of Anatomy and Clinical Anatomy, University of Zagreb School of Medicine, Zagreb, Croatia; The first two authors contributed equally.

## Abstract

GABAergic cortical interneurons are important components of cortical microcircuits. Their alterations are associated with a number of neurological and psychiatric disorders, and are thought to be especially important in the pathogenesis of schizophrenia. Here, we reviewed neuroanatomical and histological studies that analyzed different populations of cortical interneurons in postmortem human tissue from patients with schizophrenia and adequately matched controls. The data strongly suggests that in schizophrenia only selective interneuron populations are affected, with alterations of somatostatin and parvalbumin neurons being the most convincing. The most prominent changes are found in the prefrontal cortex, which is consistent with the impairment of higher cognitive functions characteristic of schizophrenia. In contrast, calretinin neurons, the most numerous interneuron population in primates, seem to be largely unaffected. The selective alterations of cortical interneurons are in line with the neurodevelopmental model and the multiple-hit hypothesis of schizophrenia. Nevertheless, a large number of data on interneurons in schizophrenia is still inconclusive, with different studies yielding opposing findings. Furthermore, no studies found a clear link between interneuron alterations and clinical outcomes. Future research should focus on the causes of changes in the cortical microcircuitry in order to identify potential therapeutic targets.

Schizophrenia is a psychiatric disorder that affects up to 1% of the population and is characterized by the dysregulation of cognitive, emotional, and behavioral functions (1,2). The clinical manifestation of schizophrenia is usually categorized into three groups of symptoms: positive, negative, and cognitive. Positive symptoms are experienced during psychotic episodes and include hallucinations, delusions, and speech disorders, while negative symptoms include apathy, flattened affect, abulia, avolition, and anhedonia. Cognitive symptoms typically manifest as deficits in memory, attention, and reasoning. They are often present in the prodromal stage, long before the manifestation of the core positive and negative symptoms (2). Among these three groups of symptoms, positive symptoms best respond to pharmacotherapy, while negative and cognitive symptoms are more resistant to treatment and are the main cause of decreased quality of life in schizophrenia (3).

Schizophrenia is diagnosed based on the Diagnostic and Statistical Manual of Mental Disorders (DSM) (4) or the International Classification of Diseases (ICD) (5). The most recent versions of these diagnostic handbooks currently in use are the DSM-5 and ICD-10, with the ICD-11 being proposed to replace ICD-10 in the near future (6).

The etiology and pathogenesis of schizophrenia are still unclear, though it is generally agreed that the clinical manifestation of schizophrenia necessitates a combination of environmental and genetic factors. There are several prevailing hypotheses on the origin of schizophrenia, yet none of these completely explain all the observed clinical manifestations. Nevertheless, most of them are not mutually exclusive, which leaves open the possibility of multiple hypotheses eventually explaining the mechanisms underlying the pathogenesis of schizophrenia (2,7).

The potential role of GABAergic cortical neurons in the pathophysiology of schizophrenia is of particular interest because schizophrenia is often associated with dysfunction of cortical microcircuits (8,9). Understanding the alterations of different populations of GABAergic cortical neurons in schizophrenia could provide important insight into the clinical presentation and treatment of this disorder.

Here, we give a comprehensive overview of the possible roles of GABAergic cortical neurons in schizophrenia. In particular, we focused on molecular studies done on human brain tissue and accentuated the strength and conclusiveness of the findings, which was not done in previous reviews on this topic. We also evaluated the different hypotheses on its etiology and pathogenesis in relation to the dysfunction of GABAergic neurons.

## Neuroanatomical background of schizophrenia

Research on the neuroanatomical background of schizophrenia is vast; however, the exact regions and functional circuits affected are still somewhat contentious. The most consistent finding in postmortem and *in vivo* studies is a relatively generalized reduction in brain volume, predominantly attributed to a reduction in gray matter of the cerebral cortex (10,11). Nevertheless, the overall thinning of the cerebral cortex does not adequately explain the typical clinical manifestations of schizophrenia, and evidence points to more subtle subcellular abnormalities being the main driving force behind the cognitive disturbances (9,12-16). Furthermore, even though the extent to which the white matter is affected varies between studies, white matter abnormalities in schizophrenia are still frequently reported. Most studies point to disrupted white matter integrity and demyelination. Nevertheless, the exact impact of white matter lesions on the pathogenesis and overall clinical presentation of schizophrenia is still being studied (17-21). Overall, the gross anatomical changes in schizophrenia are largely non-specific and are likely merely a consequence of underlying microcircuitry alterations.

Despite the lack of specificity regarding gross morphological changes in schizophrenia, the majority of studies studies suggest that the most affected regions of the brain are the prefrontal cortex (PFC), temporal lobe, and basal nuclei.

**Figure 1:**
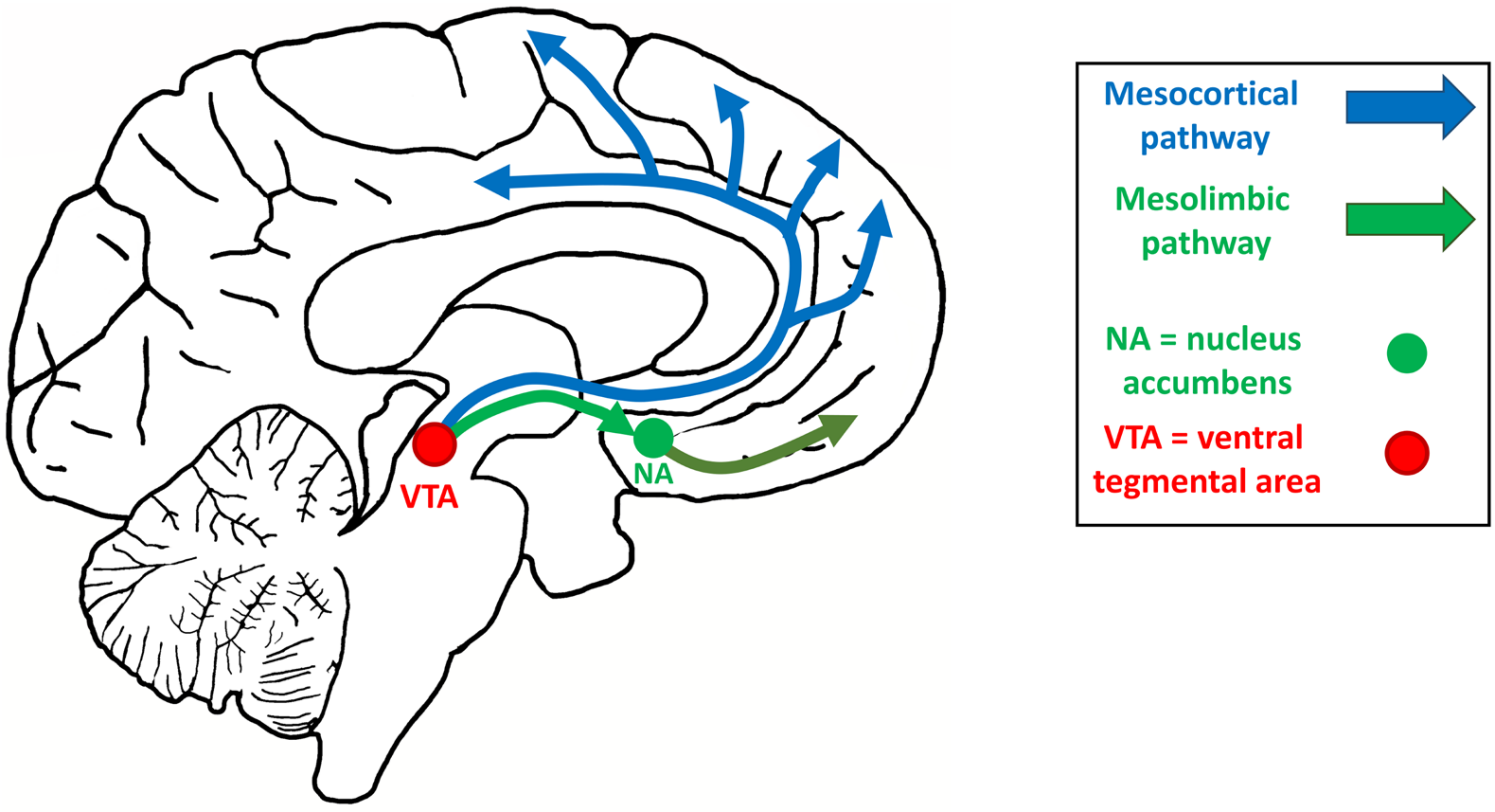
Dopaminergic pathways altered in schizophrenia – mesocortical pathway (blue) and mesolimbic pathway (light green). The origin of both pathways – the ventral tegmental area is shown as a red circle, while the light green circle represents the nucleus accumbens (NA), which is part of the ventral striatum. The dark green arrow represents projections from the NA.

The most intriguing of these is the PFC. The PFC is generally divided into two distinct functional parts – the lateral PFC (LPFC) and the ventromedial (vmPFC) or orbitomedial PFC (omPFC). The term vmPFC is sometimes used partially or completely synonymously with the term orbitofrontal cortex (OFC). The LPFC is further divided into the dorsolateral (DLPFC) and ventrolateral (VLPFC) prefrontal cortex. The LPFC is crucial for the integration of higher cognitive functions (executive functions), such as decision making and working memory, while the vmPFC is involved in the control of emotions and motivation (22). The PFC receives rich dopaminergic innervation via the mesocortical pathway, which originates in the ventral tegmental area (VTA) of the mesencephalon (Figure 1) (23). The overall effect of dopamine on prefrontal cortical neurons is predominantly inhibitory. However, it modulates the activity of cortical neurons in the PFC through at least three major modes of action (9). The first is via direct innervation of pyramidal neurons, which enables dopaminergic regulation of cortico-thalamic, cortico-striatal, and cortico-cortical projections from the PFC. The second is via non-synaptic dopamine neurotransmission, while the third is via the innervation of local circuit non-pyramidal neurons (GABAergic interneurons). This last and particularly significant mode of action enables indirect regulation of PFC projection pathways via feed-forward inhibition. Alterations in the dopaminergic innervation of the PFC via the mesocortical pathway are thought to be important in the pathophysiology of schizophrenia. The changes in the LPFC have been connected to cognitive (DLPFC) and negative (VLPFC) symptoms in schizophrenia (24). Interestingly, dysconnectivity between the cerebellum and the DLPFC has been associated with negative symptom severity (25). Furthermore, the thinning of the left medial OFC was associated with the severity of negative symptoms (26), while vmPFC dysfunction was more strongly associated with positive symptoms (24). In addition, the hypoactivity of the anterior cingulate cortex (ACC), which is often considered a functional extension of the vmPFC, was associated with the presence of negative symptoms (27-29). Alterations to von Economo neurons, a highly specialized class of projection neurons located in the ACC, have also been described (30-32).

Besides the mesocortical pathway, the mesolimbic pathway is also affected in schizophrenia. This pathway also originates in the VTA; however, its synaptic targets are located in the ventral striatum (part of the basal nuclei), which includes the nucleus accumbens and olfactory tubercle (Figure 1). In contrast to the mesocortical pathway, which is involved in the regulation of executive functions, the mesolimbic pathway is involved in aversion-related and reward-related cognition (positive reinforcement, pleasure response to stimuli, and incentive salience) (23). The ventral striatum has been associated with both positive and negative symptoms in schizophrenia (24).

The medial portion of the temporal lobe contains the hippocampus, which is usually significantly reduced in size in schizophrenia. The hippocampus and its adjacent structures are involved in short-term memory consolidation, and their dysfunction in schizophrenia can explain poor memory retrieval and some of the positive symptoms. Another important part of the temporal lobe affected in schizophrenia is the superior temporal gyrus, which is involved in language comprehension, auditory processing, and self-monitoring. Its cortico-cortical projections form part of the temporal-frontal-parietal network involved in language production and interpretation. Cortical thinning in the superior temporal gyrus is associated with the severity of positive symptoms, particularly thought disturbances and auditory hallucinations (33). However, some studies showed a less convincing association between the superior temporal gyrus and positive symptoms (24).

In general, reduced blood flow in the PFC and striatum observed on functional MRI is particularly common in patients with prominent negative and cognitive symptoms. Such patients typically experience a prolonged prodromal period, characterized by inadequate social functioning, before the onset of positive (psychotic) symptoms (23).

Overall, numerous studies investigated the neuroanatomical abnormalities in schizophrenia and their relation to the specific symptoms (24,25,27-29,33). An overview of these findings is shown in Table 1. Most of the available data refer to neuroimaging correlation studies, which typically do not determine whether the relationships are causal to the disorder, or whether they are compensatory processes or secondary phenomena.

**Table 1:** Overview of the affected anatomical regions in schizophrenia and their relation to the clinical presentation of the disorder. The level of association with certain groups of symptoms is shown in parentheses (data extrapolated from 24,25, 27-29,33)

Anatomical region affected in schizophrenia	Connection to clinical presentation of schizophrenia
Dorsolateral prefrontal cortex	cognitive symptoms (moderate association)
Ventrolateral prefrontal cortex	negative symptoms (moderate association)
Ventromedial prefrontal cortex	positive symptoms (moderate association) negative symptoms (inconclusive)*
Ventral striatum (nucleus accumbens)	negative symptoms (moderate association) positive symptoms (weak association)
Hippocampus	positive symptoms (weak association)
Amygdala	positive symptoms (weak association)
Anterior cingulate cortex	negative symptoms (inconclusive)†
Superior temporal gyrus	positive symptoms (inconclusive)*
Cerebellum	negative symptoms (inconclusive)†

*only some studies showed clear association, while others did not show clear association.

†the number of studies demonstrating a clear association was small.

## Etiology and pathogenesis of schizophrenia

The hypotheses explaining the etiology and pathogenesis of schizophrenia can be grouped into two categories – the hypotheses involving altered levels of different neurotransmitters (dopamine, GABA, and glutamate dysfunction) and the hypotheses involving reduced cortical synaptic connectivity.

Among the hypotheses related to neurotransmitter dysfunction, the most prevalent is the dopamine hypothesis. This hypothesis suggests that the main cause of psychotic symptoms in schizophrenia is excessive dopamine D2 receptor activation. It is supported by the significant efficacy of D2-like-receptor antagonists in the treatment of psychotic symptoms. This hypothesis also has a strong neuroanatomical basis, since the PFC, cingulate cortex, and medial temporal cortex, which are all extensively affected in schizophrenia, receive particularly strong dopaminergic innervation (2).

The next most prominent hypothesis is the one involving glutamate dysfunction. This hypothesis explains the etiology of both positive and negative symptoms of schizophrenia through the dysfunction of N-methyl-D-aspartate (NMDA) glutamate receptors. It is supported by ketamine (an NMDA receptor blocker) causing schizophrenia-like symptoms in pharmacological models of schizophrenia. Most models suggest a hypofunction of NMDA receptors, which could explain some of the negative and cognitive symptoms. Interestingly, the expression of the NR2D subtype of NMDA receptors in schizophrenia is increased. NR2D receptors in schizophrenia are characterized by hyperexcitability, probably as a compensatory response to reduced cortical activity. This particularly affects the stimulatory input of cortical GABAergic interneurons, thus impacting feedback inhibition in cortical circuits. Such a dysfunction of NMDA receptors is particularly prevalent in the PFC (2).

The GABA hypothesis suggests that schizophrenia occurs due to alterations in the GABAergic cortical networks. Possible mechanisms include altered GABA synthesis and re-uptake. Once again, such dysfunctions are most prominent in the PFC. Newer models attempted to integrate the GABA hypothesis with NMDA hypofunction (2). These models explain the altered cortical activity in schizophrenia by a disbalance between GABAergic and glutamatergic activity. Such a disbalance could cause instability within cortical microcircuits and lead to impaired cortical functioning, consistent with the negative and cognitive symptoms (2).

According to the disconnection hypothesis, rather than by a disbalance of particular neurotransmitters, the pathogenesis of schizophrenia can be explained primarily by reduced or dysfunctional synaptic connectivity between different cortical areas. These changes in synaptic connectivity could disproportionately affect the mesocortical pathway involving the PFC. Synaptic dysfunction impacts both local circuit neurons (typically GABAergic interneurons) and projections neurons (typically glutamatergic pyramidal neurons), with changes in microcircuits as well as in cortico-cortical and cortico-subcortical networks (34,35). Cortical connectivity could be greatly affected by structural or functional alterations to specific neuron classes. The disconnection hypothesis is also particularly interesting because it is in line with the neurodevelopmental model and the two-hit hypothesis on the pathogenesis of schizophrenia (2).

**Figure 2:**
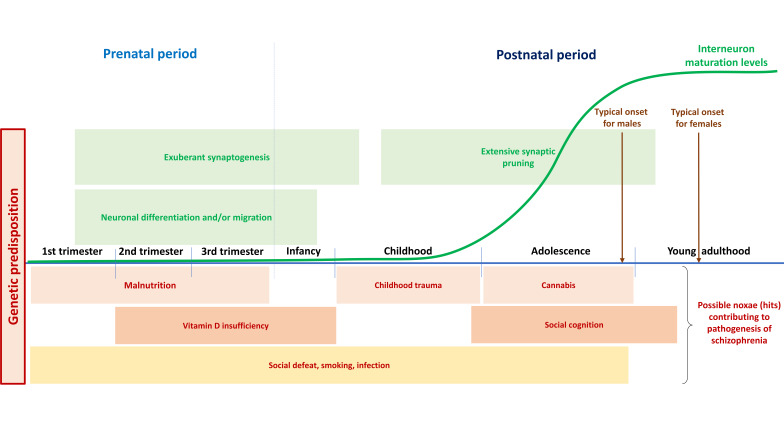
The multiple-hit neurodevelopmental model of schizophrenia. The x-axis represents the different prenatal and postnatal life periods during which certain hits (shown below the axis) can affect normal developmental processes (shown above the axis).

The neurodevelopmental model of schizophrenia proposes that the first pathological events in the brain occur long before the onset of the symptoms (7,36-39). Numerous studies demonstrated a correlation between perinatal events (eg, infection in pregnancy, placental insufficiency) and the occurrence of schizophrenia later in life. In this model, at least two hits (noxae) are necessary for the clinical manifestation of schizophrenia. The first hit occurs during the early development of the brain, either due to genetic or prenatal environmental factors. The second hit occurs during postnatal brain development. The examples of such adverse events include infectious agents, social factors (eg, social defeat – related to the negative experience of being excluded from a majority group; and social cognition – people’s perception of themselves and other individuals), and substance abuse (36). In the two-hit model, the clinical presentation of schizophrenia becomes apparent only after the second hit occurs. Certain hits may occur only during certain neurodevelopmental windows. These hits can affect the migration and maturation of neurons in critical regions of the brain, and their effects become apparent when the development of associated functions is most pronounced (Figure 2). The neurodevelopmental model does not exclude the possibility of more than two hits occurring – this is usually referred to as the multiple-hit hypothesis (36).

## Cortical interneurons in schizophrenia

Initially, research on neuropathology in schizophrenia mainly focused on cortical pyramidal neurons. The changes to pyramidal neurons were subtle, and included reduced arborization and synaptic connectivity. There was also a reduced number of specific membrane receptors, with GABA receptors being among the most affected. The latter alterations suggested that the pathological changes in pyramidal neurons might be related to the dysfunction of GABAergic cortical neurons (cortical interneurons). Therefore, research interest has recently shifted to cortical interneurons, which regulate pyramidal neuron activity in cortical microcircuits.

There are numerous studies on cortical interneurons and their alterations in schizophrenia, in both animals and humans. We reviewed the data from neuroanatomical and histological studies that analyzed different populations of cortical interneurons in postmortem human tissue from patients with schizophrenia and adequately matched controls. We focused on the studies using the following methodologies: immunohistochemistry, RNA *in situ* hybridization, and real-time polymerase chain reaction. Research revealed that even the alterations of cortical interneurons were relatively subtle and affected only certain interneuron populations. However, there is a large number of contradicting studies, and certain changes in cortical microcircuitry are less supported than others. Many of these discrepancies could be attributed to differences in methodology, the use of animal models, prolonged use of pharmacotherapy, as well as differences in the postmortem delay of the analyzed brain. Furthermore, the predominance of different types of symptoms in schizophrenia may be related to alterations in different interneuron populations.

**Figure 3:**
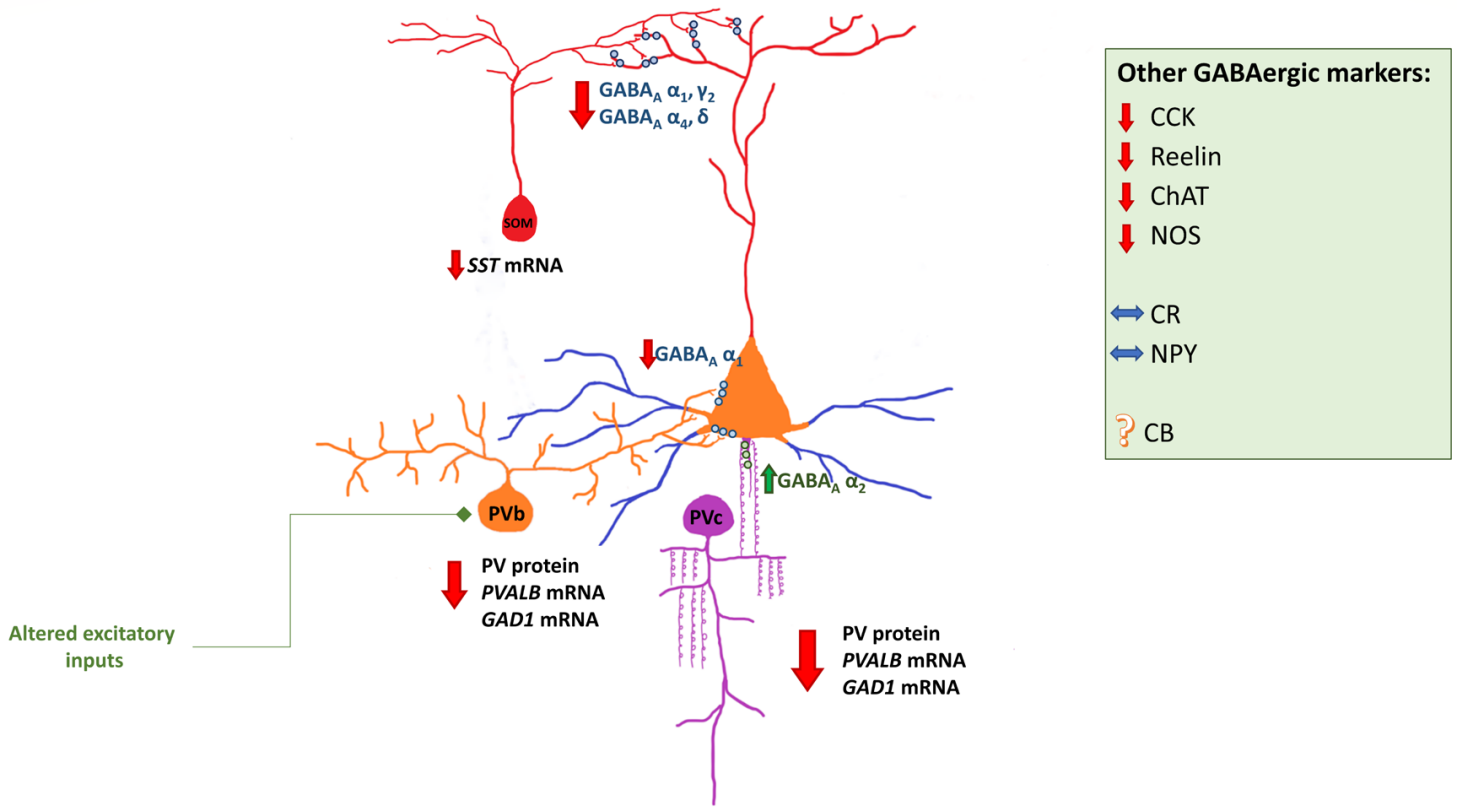
The alterations of cortical interneurons in schizophrenia. The synaptic targets of different interneuron types are shown on the pyramidal neuron in the center. Somatostatin-positive Martinotti cells (SOM) target the pyramidal neurons’ apical dendrites, parvalbumin-positive basket cells (PVb) target the somata, while the parvalbumin-positive chandelier cells (PVc) target the axon initial segments. Alterations (or lack thereof) of other common GABAergic markers are shown in the framed box on the right: cholecystokinin (CCK), reelin, choline acetyltransferase (ChAT), nitric oxide synthase (NOS), calretinin (CR), neuropeptide Y (NPY), and calbindin (CB).

The three most consistent findings among all of these studies are a general decrease in *GAD1* (glutamate decarboxylase; GAD67) mRNA expression, a decrease in *SST* (somatostatin) mRNA expression, and a decrease in parvalbumin (PV) expression and/or PV^+^ neuron density (Table 2 and Figure 3).

**Table 2:** Changes in specific GABAergic interneuron populations in schizophrenia

Interneuron population	Alteration in schizophrenia (method used)	Brain region analyzed	Relevant studies
Somatostatin	decreased expression of *SST* mRNA (ISH) reduced number of SOM^+^ neurons (IHC)	DLPFC subiculum entorhinal cortex hippocampus	Nakatani et al 2006, Hashimoto et al 2008a, Morris et al 2008, Konradi et al 2011, Wang et al 2011
Parvalbumin	decreased expression of PV protein (IHC) and/or reduced number of PV^+^ neurons (IHC)	DLPFC subiculum entorhinal cortex inferior colliculus hippocampus	Beasley and Reynolds 1997, Konradi et al 2011, Wang et al 2011, Chung et al 2016, Kilonzo et al 2020, Kalus et al 1997, Shepard et al 2019, Woo et al 1997
Calbindin	inconclusive (IHC, ISH, RT-PCR)	DLPFC temporal cortex striatum subiculum entorhinal cortex	Daviss and Lewis 1995, Benes et al 1998, Holt et al 1999, Iritani et al 1999, Takahashi et al 2000, Chance et al 2005, Fung et al 2010, Wang et al 2011
Calretinin	inconclusive (IHC, RT-PCR)	DLPFC striatum	Daviss and Lewis 1995, Woo et al 1997, Hashimoto et al 2003, Adorjan et al 2020
Cholecystokinin	decreased expression of *CCK* mRNA (ISH, RT-PCR)	DLPFC	Hashimoto et al 2008a, Fung et al 2010
Neuropeptide Y	inconclusive (IHC, RT-PCR)	DLPFC	Ikeda et al 2004, Hashimoto et al 2008a, Fung et al 2010
Nitric oxide synthase	reduced number of NOS^+^ neurons (IHC)	striatum	Fritzen et al 2007
Reelin	decreased expression of *RELN* mRNA (RT-PCR)	DLPFC	Guidotti et al 2000

*Abbreviations: *SST* – somatostatin mRNA; SOM – somatostatin protein; CCK – cholecystokinin; NOS – nitric oxide synthase; *RELN* – reelin; ISH – *in situ* hybridization; IHC – immunohistochemistry; RT-PCR – reverse transcription polymerase chain reaction; DLPFC – dorsolateral prefrontal cortex.

### Glutamate decarboxylase

Many studies found consistent alterations in *GAD1* mRNA expression in schizophrenia. *GAD1* encodes for the GAD67 protein, which is located primarily in the neuronal cell body and synthesizes GABA for numerous metabolic purposes, including neuroprotection and oxidative stress regulation (40,41). Many studies, using various techniques on both animal models and postmortem human tissue, consistently found significantly decreased *GAD1* mRNA expression in the PFC in schizophrenia (42-58). A similar decrease in *GAD1* expression was also found in other cortical areas, such as the visual cortex, hippocampus, ACC, motor cortex, and cerebellum (45,59-62). Some studies suggested that the overall reduction in *GAD1* mRNA expression in schizophrenia might be predominantly due to a selective reduction in a certain subset of cortical interneurons, rather than due to a generalized reduction affecting all interneuron populations (8).

Interestingly, *GAD2* mRNA expression in schizophrenia appears to be largely unaltered or only slightly decreased (42,45,58). *GAD2* encodes for the GAD65 protein, which is located in the axon terminals and is primarily involved in GABA synthesis for neurotransmission (40,41).

Since both the *GAD1* and *GAD2* genes are important for the production of GABA, this raises the question why only *GAD1* expression is altered. One explanation might be that *GAD2* is less expressed in healthy brain tissue compared with *GAD1*. This means that it could be more difficult to detect subtler changes in *GAD2* expression. Another explanation is the fact that GAD67 (the product of *GAD1*) is predominantly located in the soma and involved in metabolic production of GABA, while GAD65 (the product of *GAD2*) is located in the axon terminal and is involved in GABA synthesis for neurotransmission. This might suggest that in schizophrenia the neuroprotective role of GABA is more affected than GABA transmission. This is also in line with the selective loss of certain interneuron populations, such as SOM^+^ and PV^+^ cells, which seems to occur in this disorder.

Besides the described alterations to GABAergic markers, the expression of certain subtypes of GABA receptors, located predominantly on pyramidal neurons, also appears to be altered in schizophrenia (Figure 3) (8).

### Somatostatin and calbindin

Besides *GAD1* expression, there is additional convincing evidence for the alterations of *SST* mRNA expression in schizophrenia. Multiple studies demonstrate a significant decrease in *SST* expression in the PFC and hippocampus in schizophrenia (42,63-66). There is also evidence of a reduced number or density of somatostatin (SOM^+^) cells (63,66).

Unlike that on SOM, research on calbindin (CB) in schizophrenia yielded inconclusive results, with different authors finding increased, decreased, or unchanged levels of CB protein or mRNA (66-73). Several studies demonstrated a reduction in *SST* expression, but did not demonstrate a reduction in CB expression (66,73,74).

This discrepancy between SOM and CB alteration in schizophrenia is particularly interesting. In the human PFC, SOM and CB are expressed in highly overlapping interneuron populations – up to 70% of CB^+^ interneurons co-express SOM and up to 50% of SOM^+^ interneurons co-express CB (75). Therefore, the discrepancies in the expression of SOM and CB in schizophrenia point to a very selective change in the non-overlapping SOM and CB interneuron subpopulations. This could be especially notable because SOM and CB are predominantly co-expressed by interneurons in the supragranular cortical layers (layers II and III). The non-overlapping subpopulations are located predominantly in the infragranular layers (layers V and VI) and may have different synaptic targets with involvement in different cortical microcircuits (75). Nevertheless, it is also possible that SOM as a neuromodulator is differently affected in schizophrenia than CB as a calcium-binding protein.

### Parvalbumin

Even though PV^+^ neurons have probably been the most studied interneuron population in schizophrenia, there is still no consensus on how exactly PV^+^ cells are altered in this disorder.

Most research suggests a decrease in PV protein or *PVALB* mRNA levels (47,63,66,76-78). However, some animal models and some human studies found the levels of PV protein to be elevated (79,80). A smaller number of studies revealed no significant alterations in PV^+^ cell density (47). Some studies claimed that lower PV levels in schizophrenia reflected only a decrease in PV expression in a subset of PV^+^ neurons and not an actual deficit in PV^+^ neuron numbers (76,81-84). Though these opposing claims could be explained by methodological and regional differences between studies, it is difficult to determine whether this is truly the case. Possibly, different cohorts exhibited different changes to the PV^+^ interneuron population, some resulting in cell loss and others resulting in decreased PV expression. The overall findings could also be influenced by the severity and type of symptoms, as well as the pharmacotherapy the patients were exposed to during the course of their lives.

Some studies demonstrated that a reduction in PV^+^ neurons also confirmed a reduction in *Wisteria floribunda* agglutinin (WFA^+^) perineuronal nets (PNNs) in schizophrenia (83), which are predominantly related to a certain subpopulation of PV^+^ neurons (85). Such findings suggested that PV^+^ neuron loss in schizophrenia could be rather selective, targeting only specific neuronal subpopulations (86). However, in the amygdala and the entorhinal cortex, a reduction in the number of PNNs was not accompanied by a reduction in PV^+^ cell density (83). WFA^+^ PNNs are also found around certain pyramidal neurons – this means that reductions in the number of WFA^+^ PNNs and PV^+^ cells are not necessarily always related to each other.

### Calretinin

Unlike the alternations in SOM^+^ and PV^+^ interneuron populations, most research found no significant alterations in the CR^+^ cortical interneuron population in schizophrenia (47,69,81,87). However, some studies found a reduction in CR^+^ neurons in subcortical structures, such as the striatum (88). Together, CR, PV, and SOM likely mark the vast majority of GABAergic cortical neurons, at least in the human PFC (89). Out of these three large non-overlapping populations, CR^+^ neurons are the most numerous in the primate brain (90). It is, therefore, intriguing that this is the only interneuron population that is largely unaffected in schizophrenia. Furthermore, CR^+^ neurons are relatively unaltered in most other neuropsychiatric or neurodegenerative disorders, such as Alzheimer’s disease and depression (91). CR is a calcium-binding protein that protects neurons from calcium cytotoxicity, and this neuroprotective role might provide CR^+^ neurons with a unique resistance to various noxae (92). Nevertheless, this would still not explain why other neuron populations expressing different types of calcium-binding protein, particularly PV, are significantly affected in schizophrenia and other disorders. Another explanation is that CR^+^ neurons, as a vital component of cortical microcircuits in the human brain, are significantly altered only in rare and/or extremely severe disorders.

### Other interneuron markers

Significant findings regarding other interneuron markers include reduced expression of *CCK* mRNA (cholecystokinin) (42,73), decreased expression of *RELN* mRNA (reelin) (45), and a reduced number of nitric oxide synthase (NOS^+^) neurons (93). Studies on neuropeptide Y were less conclusive and demonstrated no changes or a very subtle decrease in its expression or cell number (42,73,94).

## Different developmental origins of interneuron populations affected in schizophrenia could be in line with the neurodevelopmental model of schizophrenia

The differences in alterations between different interneuron populations could also be explained by the distinct developmental origin of CR^+^ neurons compared with SOM^+^ and PV^+^ neurons (95). Whereas CR^+^ neurons originate from the caudal ganglionic eminence and the dorsal proliferative zones (in primates), SOM^+^ and PV^+^ neurons originate from the medial ganglionic eminence (MGE) and preoptic area (POA) (89). If interneuron alterations in schizophrenia are caused by an early hit during interneuron development, this could mean that pathological changes primarily occur in the MGE and POA. However, interneuron alterations could also be caused by a later hit occurring during adolescence, when most interneuron maturation occurs. If this is the case, PV^+^ and SOM^+^ neurons might simply be more vulnerable to the pathological changes occurring in schizophrenia or to the specific hits typically occurring in this life period (eg, cannabis abuse). Therefore, both an early and a late hit possibility could be in line with the neurodevelopmental model of schizophrenia and the multiple-hit hypothesis. Moreover, multiple hits to specific interneuron populations at different life stages are not mutually exclusive.

## Conclusions

In conclusion, specific populations of GABAergic cortical interneurons are selectively affected in schizophrenia. Changes in the somatostatin interneuron population are the most substantiated in the literature, followed by alteration of parvalbumin neurons. Calretinin neurons seem to be largely unaltered, at least in the cerebral cortex. The changes in selective interneuron populations are most pronounced in specific cortical regions, particularly the PFC, where they likely have highly specific effects on the cortical microcircuitry. Nevertheless, the question remains whether the described changes in schizophrenia are part of the underlying pathophysiological mechanism that contributes to the clinical manifestation of the disorder, or whether they are a consequence of other underlying mechanisms. Future research should focus on determining the exact causes of these changes in the cortical microcircuitry in order to identify potential therapeutic targets. Such research could be especially beneficial if we want to better understand the pathophysiology and treatment of negative and cognitive symptoms.
